# Relationship between symptoms and both stage of change in adopting a healthy life style and quality of life in patients with liver cirrhosis: a cross-sectional study

**DOI:** 10.1186/s12955-021-01787-9

**Published:** 2021-05-17

**Authors:** Myung Kyung Lee, Woo Jin Chung

**Affiliations:** 1 College of Nursing, Research Institute of Nursing Science, Kyungpook National University, 41944, 680 Gukchabosangro, Jung-gu, Daegu, South Korea; 2 Department of Internal Medicine, Keimyung University School of Medicine, Daegu, South Korea

**Keywords:** Symptom, Exercise, Diet, Quality of life, Liver cirrhosis

## Abstract

**Background:**

Previous studies of patients with liver cirrhosis have not considered the broad range of symptoms or the association between healthy behavior and quality of life. The purposes of this study were to examine the association between symptoms and adopting exercise and consuming fruits and vegetables and to identify factors associated with quality of life in patients with liver cirrhosis.

**Methods:**

This cross-sectional study enrolled 91 consecutive patients with liver cirrhosis in one tertiary general hospital in South Korea between February 2016 and January 2017. Each study participant completed a self-administered questionnaire that measured symptom, stage of change in engaging in exercise and consumption of fruits and vegetables, and the Korean version of the 36-item Short-Form Health Survey. Multivariate ordinal logistic regression analysis and multiple regression models was used, respectively, to examine the association between each symptom with stage of change in engaging in exercise and consumption of fruits and vegetables and to evaluate factors affecting quality of life.

**Results:**

Experiencing nausea was associated with more readiness for change in engaging in exercise, but experiencing shortness of breath was associated with less readiness for change in engaging in exercise. Experiencing right upper quadrant pain was associated with more readiness for change in engaging in consumption of fruits and vegetables. Muscle cramps, anorexia, right upper quadrant pain and body pain, itching, ascites or edema, bruising, and change in appearance negatively affected quality of life.

**Conclusions:**

The results suggest that the types of symptoms experienced by a patient with liver cirrhosis hinder or promote the patient’s adoption of exercise and dietary behavior. Experiencing symptoms may negatively affect quality of life. Caregivers should provide supportive care to patients with liver cirrhosis, which includes assessing and managing symptoms to improve quality of life.

## Background

Deaths from viral hepatitis are much more frequently due to progression to liver cirrhosis (LC; 720,000 deaths worldwide in 2015) than hepatocellular carcinoma (470,000 deaths worldwide in 2015) [1]. As LC progresses, patients may experience progressive deterioration of liver function and accompanying signs and symptoms. Thus, most individuals with LC report multiple symptoms or complications [2], which can lead to a significant decline in quality of life (QOL) [3]. A better QOL is a predictor of survival in patients with LC [4]; thus, management of symptoms is critical for improving the QOL of patients with LC. Previous studies have examined the association between QOL and gastrointestinal symptoms, ascites, fatigue, and laboratory data [3, 5] in patients with LC, but they have not considered the wide range of symptoms and complications experienced by these patients. Indeed, even with self-reported data, it is not possible to fully understand LC patients’ QOL.

In patients with LC, adopting healthy behaviors is a significant predictor of morbidity and survival [6], and it is generally known to improve the QOL of patients with chronic diseases [7]. The prevalence of deteriorating physical functioning is high and leads to a poor prognosis in cirrhosis. In response to this, it is recommended that all patients with cirrhosis work to improve their QOL [8], particularly by performing regular exercise [9] and healthy eating [10].

Although exercising is known to improve QOL in LC patients, patients with LC are reported to have low levels of physical activity, with 76% of their day spent in a sedentary state [11]. In addition, malnutrition is one of the most universal problems of LC and is related to an increased risk of worsening disease progression and mortality [6]. Dietary guidelines for patients with LC indicate that fruits and vegetables (F&V) and whole grains, as well as certain types of proteins in appropriate quantities, are good foods for individuals with the condition [12]. Recommendations regarding protein intake given in general guidelines cannot be applied to patients with LC because the protein intake of such patients should be carefully adjusted based on liver function, ascites, and blood ammonia levels [13]. On the other hand, the guideline for the intake of other foods such as F&V can be suited to patients with liver diseases. With regard to the adoption of a healthy diet, to the best of our knowledge, few studies have identified topics such as the adoption of an appropriate intake of F&V in patients with LC. Exploration of the relationship between symptoms and adoption of healthy behaviors could provide basic information to help devise appropriate strategies for promoting healthy behaviors in patients with a particular symptom.

The transtheoretical model (TTM) is an integrative, biopsychosocial model used to conceptualize the process of intentional behavior change [14]. Stage of change (SOC) lies at the center of the TTM [14, 15] and is widely used as an outcome in diet and physical activity interventions [16]. Although progression through the SOC can occur in a linear fashion, a nonlinear progression is common. Often, individuals recycle through the stages or regress to earlier stages from later ones. SOC items characterize participants into five stages: precontemplation (not considering change), contemplation (considering change and potential benefits), preparation (planning and problem solving for change strategies), action (implementing change strategies), and maintenance. This study aimed to assess the use of the SOC as an outcome for diet and exercise behavior for adults with LC.

Researchers have reported that factors associated with poor QOL of patients with LC are sociodemographic characteristics, including age, sex, paid employment, sex life, social life, symptom presence (i.e., pain and muscle cramps, ascites, pruritus), and perceived health problems [17]. Prior studies indicated that exercise improves physical frailty and QOL in patients with chronic liver disease [18]. The increased VO_2max_ and functional capacity benefits from performing aerobic exercise [19] and progressive resistance training [20] had a significant impact on improved QOL in liver disease patients. In addition, liver disease patients who received health empowerment education, including clinical symptoms, diet, and nutrition, were shown to have improved cognitive levels, activities of daily living, and QOL [21, 22].

With regard to symptoms and performing exercise, one study reported that LC patients commonly had impairments in maximal exercise capacity and pulmonary gas exchange abnormalities [23] and that the subsequent fatigue can alter their aerobic capacity. Although we were unable to find recent prior studies on symptoms and adherence to diet in patients with liver disease, we can predict that as compared with healthy individuals, nutritional deficiencies may occur in LC patients because of decreased dietary quantity, decreased digestive and absorption functions, changes in nutrient metabolism, increased nutritional requirements, ascites, or swelling. Thus, it is necessary to manage these patients’ diets. The diverse symptoms of patients with LC can significantly impair QOL [24]. To date, few studies on liver disease patients have dealt with symptoms, exercise and dietary behavior, and QOL together.

Healthy behaviors, such as exercise and eating F&V, are tied to the diverse systemic symptoms experienced by LC patients, which conversely hinder the adoption of these behaviors. By determining that certain symptoms experienced by LC patients are correlated with adopting healthy behaviors, we can work to improve their QOL by controlling patients’ subjective symptoms or encouraging the adoption of exercise and consuming F&V.

The aims of the present study were to examine the association between symptoms and SOC of engaging in exercise and consuming F&V and to examine the factors influencing QOL in a population of patients with LC. We propose two hypotheses: the first hypothesis is that symptoms will be differently associated with (hinder or facilitate) engaging in exercise and consuming F&V across the different types of symptom. The second hypothesis is that some symptoms will negatively affect QOL.

## Methods

This study used a cross-sectional design. The participants were recruited sequentially from one tertiary general hospital in South Korea between February 2016 and January 2017. Enrolled patients were older than 19 years, had a diagnosis of LC within the previous 2 years, and were undergoing regular treatment or follow-up. Patients were excluded if they were not physically or mentally healthy enough to complete the questionnaires or unable to understand the purpose of the study or provide informed consent.

We reviewed the hospital registries and initial assessments of patients returning to the hospital for follow-up or treatment. From this review, we identified 98 patients as eligible, and 91 of them (95%) accepted the informed consent document and completed the questionnaire. For the seven individuals who refused, the most common reason for rejecting participation was “inconvenience”. G*Power 3.1.9.7 calculated a required sample of 84, which was based on indices such as statistical analysis multiple regression, effect size f^2^ 0.21 [25], type 1 error 5%, power 80%, and nine predictors. Thus, the final sample size of 91 was acceptable.

Research staff contacted eligible patients at the ambulatory clinic and inpatient ward to explain the details of the study personally. After providing informed consent, each participant was given a packet of questionnaires in a quiet and independent meeting room, and they filled out the self-reported questionnaire in the presence of the research staff. The questionnaires collected information on sociodemographic characteristics, symptoms, SOC for adopting healthy behaviors based on the TTM [14], and QOL. The study protocol conformed with the ethical guidelines of the 1975 Declaration of Helsinki as reflected in a priori approval by the affiliated Institutional Review Boards of the hospital (2015-09-020-003) and the university (2015-87).

### Measures

#### Symptoms

Participant’s multidimensional symptom profile was measured using a self-reported scale developed in a previous study [25]. This scale assesses 18 symptoms: fatigue, muscle cramps, decrease in memory, itching, dyspepsia, anorexia, dark urine, drowsiness, dry mouth, bruising, right upper quadrant (RUQ) pain, shortness of breath (SOB) and/or dyspnea, nausea and/or vomiting, body pain, urinary difficulty, tarry stools, ascites/edema, and change in appearance. The patient rated each item in frequency, intensity, and distress on a four-point Likert-type scale (0 = *never experienced* to 3 = *extremely experienced in frequency, intensity, and distress*). The symptom score was calculated by summing the scores of frequency, intensity, and distress for each symptom. Thus, the score ranged from 0 to 162, with a higher score indicating having experienced more severe symptoms. The Cronbach’s alpha for the symptom scale in this study was 0.96.

#### Adopting healthy behaviors

Adopting healthy behaviors was evaluated from the SOC of TTM. The SOC assessments for engaging in exercise and consumption of F&V were adapted from a previous study [26], in which the measure was developed from the TTM of behavior change [14]. These measures characterized differences in the SOC of goal behaviors related to exercise and diet, from preadoption to adoption. The five items of each measure characterized participants into the following five stages: precontemplation, contemplation, preparation, action, and maintenance. Precontemplation describes an individual who is not engaged in a new behavior and has no intention of adopting the behavior in the near future. Contemplation indicates an individual who is not engaged in the behavior but is considering adopting the behavior in the next 6 months. Preparation describes an individual who has initiated some behavior changes, but the changes are not regular. Action describes an individual who is regularly engaged in the behavior but only initiated this new behavior in the past 6 months. Maintenance indicates an individual who has been regularly engaging in the behavior for more than 6 months.

Before questioning, exercise was defined as “brisk walking, cycling, swimming, mountain climbing, or another form of exercise that makes your heart pound, or makes you break out in a sweat, and is not part of your normal job activity”; F&V servings were defined as “one serving of vegetables is equal to 1/2 cup of cooked (or parboiled) vegetable, 1 cup raw vegetable or vegetable juice, and 2 cups leafy salad greens” and “one serving of fruits is 1 cup fruit or 1/2 cup of fruit juice (orange juice, etc.) or 1/3 cup of a fruit juice blend”.

**Fig. 1:**
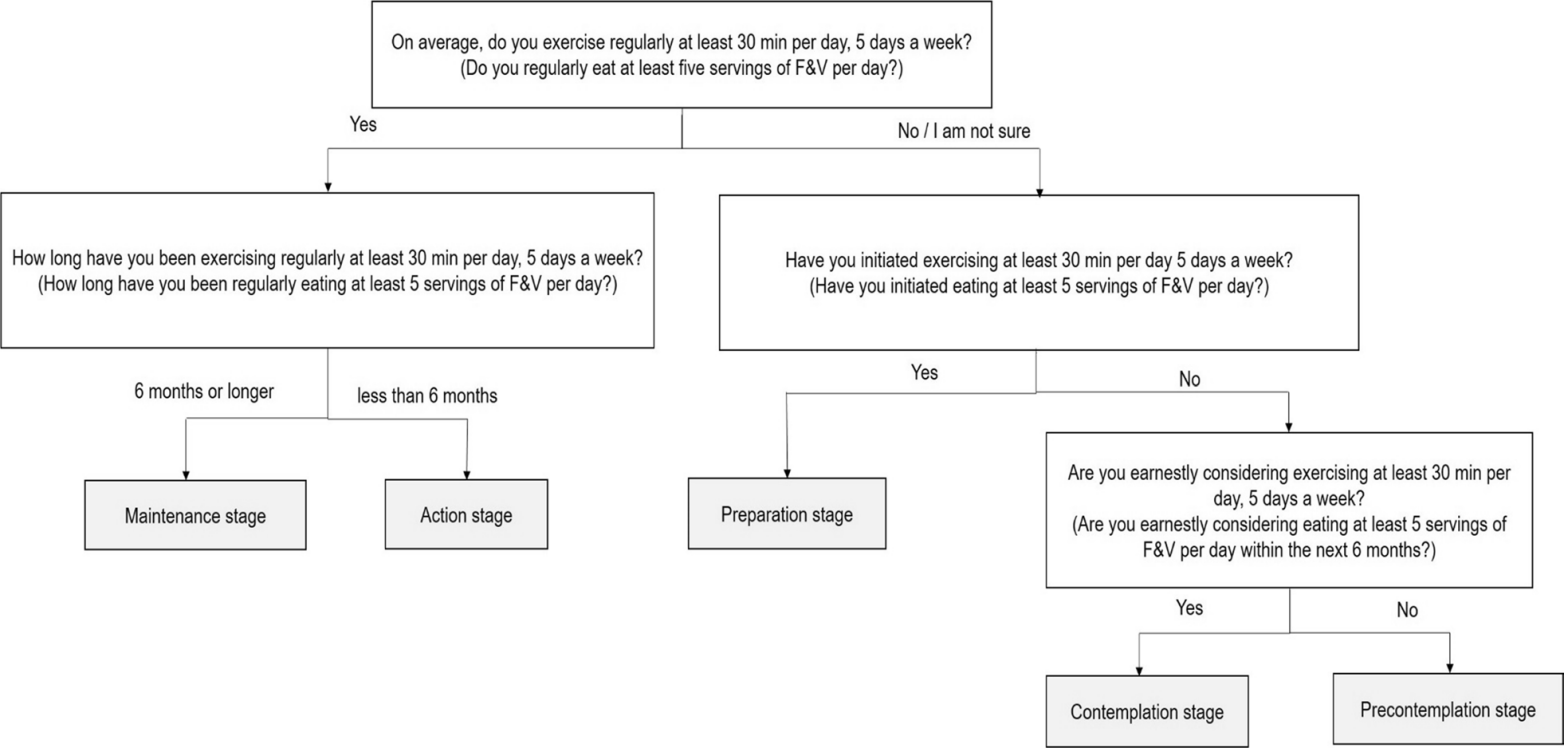
Stage-of-change assessment

Participants were then asked, “On average, do you exercise regularly at least 30 min per day, 5 days a week and do you regularly eat at least five servings of F&V per day?” [27]. If the participant responded “yes”, the next questions were posed: “How long have you been exercising regularly at least 30 min per day, 5 days a week and regularly eating at least five servings of F&V per day?” The responses were “less than 6 months” (action stage) and “6 months or longer” (maintenance stage). If a patient replied “no” or “I am not sure” to the initial question, they were asked the following question: “Have you initiated exercising at least 30 min per day, 5 days a week and eating at least five servings of F&V per day?” The responses were “yes” (preparation stage) and “no”. If the response was “no”, the patient was asked the following question: “Are you earnestly considering exercising at least 30 min per day, 5 days a week and eating at least five servings of F&V per day within the next 6 months?” The possible responses were “yes” (contemplation stage), “no”, and “I am not sure” (precontemplation stage). Precontemplation, contemplation, and preparation were classified into “not engaging in exercise or consumption of F&V”, and action and maintenance were classified into “engaging in exercise or consumption of F&V” (Fig. 1). Each SOC was scored as precontemplation = 1, contemplation = 2, preparation = 3, action = 4, and maintenance = 5, indicating higher score means more readiness for change.

#### QOL

QOL was assessed using the validated Korean version of the Medical Outcomes Study 36-item Short-Form Health Survey (SF-36) [28]. This questionnaire consists of 36 items among eight subscales: physical functioning, role limitations due to physical health problems, role limitations due to emotional problems, social functioning, body pain, general health, vitality, and mental health. Among the subscales of the SF-36, we considered only the functioning scales in this study; thus, subscales measuring general health, mental health, and vitality were excluded. In addition, because symptoms were considered independent variables, the bodily pain subscale was excluded from the dependent variable because of the problem of multicollinearity. The present study used 19 items from the following four subscales: physical functioning, role limitations due to physical health problems, role limitations due to emotional problems, and social functioning. The score on each subscale ranged from 0 to 100, with a higher score indicating better functioning; however, a higher score on role limitation indicated greater limitation. The internal consistency for the SF-36 was good (Cronbach’s alpha = 0.85) [28]. Use of the questionnaire was approved by Optuminsight Life Sciences, Inc (Lincoln, RI, USA).

### Statistical analysis

Sociodemographic characteristics and degree of dimensional symptoms were presented using descriptive statistics. First, in the univariate analyses, an independent samples *t*-test was used to examine the associations between sociodemographic parameters and total score of symptoms, and Fisher’s exact test was used to examine the associations between sociodemographic parameters and engaging in exercise and consumption of F&V. For the Fisher’s exact test, we categorized the stages of precontemplation, contemplation, and preparation into “not engaging in exercise or consuming F&V” and the stages of action and maintenance into “engaging in exercise and/or consuming F&V”. Then, because the five categories of SOCs constituted a dependent variable, we used multivariate ordinal logistic regression analysis with stepwise selection to examine the independent effect of each symptom on engaging in exercise or consumption of F&V, controlling for age, sex, marital status, having a religion, job status, monthly income, residence area, and number of family members living together. The results were presented as adjusted odds ratio (aOR) with 95% confidence interval (CI). Descriptive statistics were shown for the symptoms demonstrating significant changes according to the five SOCs in adopting exercise and consumption of F&V, and we analyzed the correlations between the SOC and symptoms using Pearson correlation coefficient. Multiple regression analysis with stepwise selection, in which the main independent variables were engaging in exercise and consumption of F&V, sociodemographic characteristics, and all symptom subscales, was used to identify the independent effect on QOL. These results are presented as beta coefficients and standard errors. For the multivariate ordinal logistic regression and multiple regression analyses, we selected independent variables based on a previous study on factors associated with poor QOL in patients with LC [17]. All statistical analyses were conducted using SAS 9.4 package (SAS Institute, Cary, NC, USA).

## Results

### Participant characteristics

Of the 91 patients, 56% were male; the mean patient age was 59 years, and 70% of patients were younger than 65 years (Table 1). Most participants were married and covered by the national health insurance system (71.4%). More than half of the patients had no job at the time of the questionnaire (59.3%), two-thirds (65.9%) lived in a metropolitan area, and about 60% were living with two or fewer family members (Table 1).

**Table 1:** Association of sociodemographic characteristics with both symptoms and SOC in engaging in healthy behavior

Sociodemographic characteristics	n (%)	Symptom	*p*	SOC in engaging in exercise		*p*	SOC in engaging in consumption of F&V		*p*
				Not engaged (precontemplation, contemplation, and preparation)	Engaged (action and maintenance)		Not engaged (precontemplation, contemplation, and preparation)	Engaged (action and maintenance)	
		Mean (SD)		n (%)	n (%)		n (%)	n (%)	
Sex									
Male	51 (56.0)	31.7 (24.8)		45 (69.23)	19 (73.08)		46 (56.8)	5 (50.0)	
Female	40 (44.0)	35.9 (24.8)	0.427	20 (30.77)	7 (26.92)	0.803	35 (43.2)	5 (50.0)	0.744
Age, years									
< 65	64 (70.3)	33.1 (23.7)		45 (69.2)	19 (73.1)		57 (70.4)	7 (70.0)	
≥ 65	27 (29.7)	34.7 (27.6)	0.788	20 (30.8)	7 (26.9)	0.803	24 (29.6)	3 (30.0)	0.999
Marital status									
No spouse	26 (28.6)	37.5 (26.0)		21 (32.3)	5 (19.2)		22 (27.2)	4 (40.0)	
With spouse	65 (71.4)	32.0 (24.3)	0.343	44 (67.7)	21 (80.8)	0.305	59 (72.8)	6 (60.0)	0.463
Practice a religion									
No	30 (33.0)	36.2 (24.5)		23 (35.4)	7 (26.9)		26 (32.1)	4 (40.0)	
Yes	61 (67.0)	32.3 (25.0)	0.483	42 (64.6)	19 (73.1)	0.472	55 (67.9)	6 (60.0)	0.724
Current job									
No	54 (59.3)	40.0 (27.7)		40 (61.5)	14 (53.8)		47 (58.0)	7 (70.0)	
Yes	37 (40.7)	24.2 (15.9)	**0.001**	25 (38.5)	12 (46.2)	0.637	34 (42.0)	3 (30.0)	0.520
Monthly household income, $									
< 2000	47 (51.6)	39.6 (28.7)		32 (49.2)	15 (57.7)		39 (48.1)	2 (20.0)	
≥ 2000	44 (48.4)	27.1 (17.8)	**0.014**	33 (50.8)	11 (42.3)	0.495	42 (51.9)	8 (80.0)	0.092
Residence									
City or town	31 (34.1)	36.0 (25.2)		20 (30.8)	11 (42.3)		27 (33.3)	4 (40.0)	
Metropolitan	60 (65.9)	32.3 (24.7)	0.510	45 (69.2)	15 (57.7)	0.333	54 (66.7)	6 (60.0)	0.730
National Health Service									
Health insurance	65 (71.4)	30.2 (21.8)		47 (72.3)	18 (69.2)		60 (74.1)	5 (50.0)	
Medical aid	26 (28.6)	42.1 (29.8)	**0.037**	18 (27.7)	8 (30.8)	0.801	21 (25.9)	5 (50.0)	0.142
Number of family members living together									
≤ 2	55 (60.4)	34.4 (26.7)		37 (56.9)	18 (69.2)		49 (60.5)	6 (60.0)	
≥ 3	36 (39.6)	32.4 (21.9)	0.706	28 (43.1)	8 (30.8)	0.346	32 (39.5)	4 (40.0)	0.999

*p* values in bold type show statistically significant results. SOC, stage of change; F&V, fruits and vegetables; SD, standard deviation

### Overall symptoms

Analysis of the 18 symptoms indicated that fatigue was the most-experienced symptom (mean [M] = 4.0, SD = 2.5), followed by muscle cramps (M = 3.2, SD = 2.4), and decrease in memory (M = 2.7, SD = 2.3), and the least experienced symptom was change of appearance (M = 0.9, SD = 1.9). The mean of the total symptom score of the participants was 33.6 (standard deviation [SD], 24.76) (Table 2).

**Table 2:** Degree of symptoms

Rank	Symptom	Mean (SD)
1	Fatigue	4.0 (2.5)
2	Muscle cramps	3.2 (2.4)
3	Decrease in memory	2.7 (2.3)
4	Itching	2.4 (2.7)
5	Dyspepsia	2.4 (2.3)
6	Anorexia	2.1 (2.5)
7	Dark urine	2.0 (2.3)
8	RUQ pain	1.9 (2.1)
9	Drowsiness	1.8 (2.2)
10	Dry mouth	1.8 (2.2)
11	Bruising	1.6 (2.1)
12	Shortness of breath/dyspnea	1.4 (1.9)
13	Nausea/vomiting	1.3 (1.9)
14	Bodily pain	1.3 (2.0)
15	Urinary difficulty	1.1 (1.8)
16	Tarry stools	1.1 (1.9)
17	Ascites/edema	1.0 (1.9)
18	Change in appearance	0.9 (1.9)
	Total symptom score	33.6 (24.8)

SD, standard deviation; RUQ, right upper quadrant

### Correlations of sociodemographic characteristics with symptoms and engaging in healthy behaviors

Unemployment (*p* = 0.001), low monthly household income (*p* = 0.014), and joining Medical Aid, the national insurance for low-income individuals in South Korea (*p* = 0.037), were correlated with more symptoms. Sociodemographic characteristics were not significantly associated with engaging in exercise and dietary behavior (Table 1).

### Independent association of symptoms with SOC in engaging in exercise and consuming F&V

The results of the multivariable analysis indicated that experiencing nausea and/or vomiting was associated with more readiness for change in engaging in exercise (aOR = 2.25, 95% CI 1.70–2.98), whereas experiencing SOB was associated with less readiness for change in engaging in exercise (aOR = 0.61, 95% CI = 0.48–0.78). In addition, experiencing RUQ pain was associated with less readiness for change in engaging in consumption of F&V (aOR = 3.16, 95% CI 2.23–4.49) (Table 3).

**Table 3:** Multivariate ordinal logistic regression: Association of symptoms with the SOC in engaging in healthy behavior

Symptom	Adjusted^a^ OR (95% CI) for higher SOC in engaging in exercise	*P*	Adjusted^a^ OR (95% CI) for higher SOC in engaging in consumption of F&V	*p*
Shortness of breath	0.61 (0.48–0.78)	< .0001	–	NS
Nausea/vomiting	2.25 (1.70–2.98)	< .0001	–	NS
RUQ pain	–	NS	3.16 (2.23–4.49)	< .0001

OR, odds ratio; SOC, stage of change; CI, confidence interval; F&V; fruits and vegetables; RUQ, right upper quadrant

^a^Adjusted for age, sex, marital status, practice a religion, job status, monthly household income, residence area, and the number of family members living together

### Correlation between symptoms and SOC in both engaging in exercise and consuming F&V

More readiness for change in engaging in exercise was correlated with lower SOB (*r* =  − 0.31, *p* = 0.003) and more severe nausea and vomiting (*r* = 0.54, *p* < 0.0001). More readiness for change in consuming F&V was correlated with more severe RUQ pain (*r* = 0.78, *p* < 0.0001) (Table 4).

**Table 4:** Correlation between SOC in engaging in exercise and both shortness of breath and nausea/vomiting, and between SOC in consuming F&V and RUQ pain

Symptom	n (%)	Shortness of breath			Nausea/vomiting			n (%)	RUQ pain		
		Mean (SD)	Correlation coefficient	*p*	Mean (SD)	Correlation coefficient	*p*		Mean (SD)	Correlation coefficient	*p*
SOC in engaging in exercise											
Precontemplation	9 (9.9)	2.2 (1.9)			1.1 (1.3)			–	–		
Contemplation	21 (23.1)	2.0 (2.5)			1.0 (1.4)			–	–		
Preparation	35 (38.5)	1.2 (1.8)			1.0 (1.6)			–	–		
Action	4 (4.4)	2.2 (2.1)			2.1 (2.6)			–	–		
Maintenance	22 (24.2)	0.4 (0.8)	− 0.31	0.003	2.1 (1.7)	0.54	< .0001	–	–	–	–
SOC in engaging in consuming F&V											
Precontemplation	–	–			–	–			0.7 (0.9)		
Contemplation	–	–			–	–			1.4 (0.6)		
Preparation	–	–			–	–			2.2 (1.7)		
Action	–	–			–	–			5.0 (3.4)		
Maintenance	–	–	–	–	–	–	–	–	7.3 (0.71)	0.78	< .0001

SD, standard error; SOC, stage of change; RUQ, right upper quadrant; F&V, fruits and vegetables

### Factors independently associated with QOL

Physical functioning was negatively associated with experiencing RUQ pain (β =  − 3.69, partial *R*^2^ = 28%, *p* < 0.0001), muscle cramps (β = -4.29, partial R^2^ = 12%, *p* < 0.0001), itching (β =  − 2.28, partial *R*^2^ = 5%, *p* = 0.006), ascites/edema (β =  − 2.98, partial *R*^2^ = 3%, *p* = 0.025), bruising (β =  − 2.44, partial *R*^2^ = 3%, *p* = 0.034), and anorexia (β =  − 1.67, partial *R*^2^ = 2%, *p* = 0.049). The linear model with these independent variables explained 53% of the total variance in physical functioning.

Social functioning was negatively associated with experiencing body pain (β =  − 5.55, partial *R*^2^ = 43%, *p* < 0.0001) and anorexia (β =  − 1.58, partial *R*^2^ = 3%, *p* = 0.033) and positively associated with being employed (β = 10.30, partial *R*^2^ = 6%, *p* = 0.003). The linear model with these independent variables explained 52% of the total variance in social functioning.

Role limitations due to physical health problems were positively associated with experiencing muscle cramps (β = 4.32, partial *R*^2^ = 24%, *p* < 0.0001) and anorexia (β = 4.42, partial *R*^2^ = 7%, *p* = 0.004) and negatively associated with being married (β =  − 19.86, partial *R*^2^ = 6%, *p* = 0.004). The linear model with these independent variables explained 37% of the total variance in role limitations resulting from physical health problems.

Role limitations due to emotional problems was positively associated with experiencing muscle cramps (β = 7.18, partial *R*^2^ = 26%, *p* < 0.0001) and change in appearance (β = 5.25, partial *R*^2^ = 26%, *p* = 0.005). The linear model with these independent variables explained 52% of total variance in role limitations resulting from emotional problems (Table 5).

**Table 5:** Factors independently associated with quality of life

Factor	Physical functioning			Social functioning			Role limitations due to physical health problems			Role limitations due to emotional problems		
	Partial *R*^2^	β (SE)	*p*	Partial *R*^2^	β (SE)	*p*	Partial *R*^2^	β (SE)	*p*	Partial *R*^2^	β (SE)	*p*
RUQ pain	0.28	− 3.69 (1.04)	< .0001	–	–	NS	–	–	NS	–	–	NS
Muscle cramps	0.12	− 4.29 (0.90)	< .0001	–	–	NS	0.24	4.32 (1.40)	< .0001	0.26	7.18 (1.44)	< .0001
Itching	0.05	− 2.28 (0.85)	0.006	–	–	NS	–	–	NS	–	–	NS
Ascites/edema	0.03	− 2.98 (1.13)	0.025	–	–	NS	–	–	NS	–	–	NS
Bruising	0.03	− 2.44 (1.01)	0.034	–	–	NS	–	–	NS	–	–	NS
Anorexia	0.02	− 1.67 (0.84)	0.049	0.03	− 1.58 (0.73)	0.033	0.07	4.42 (1.27)	0.004	–	–	NS
Change in appearance	–	–	NS	–	–	NS	–	–	NS	0.26	5.25 (1.84)	0.005
Bodily pain	–	–	NS	0.43	− 5.55 (0.91)	< .0001	–	–	NS	–	–	NS
Being married	–	–	NS	–	–	NS	0.06	− 19.86 (6.76)	0.004	–	–	NS
Having a job	–	–	NS	0.06	10.30 (3.42)	0.003	–	–	NS	–	–	NS
Model *R*^2^	0.53			0.52			0.37			0.52		

SE, standard error; RUQ, right upper quadrant; NS, not significant

The model included stage of change in engaging in exercise and consumption of vegetables, sociodemographic characteristics, and all subscales of symptoms as the main independent variables. A higher score on functioning indicates better functioning, and a higher score on role limitation indicates more severe role limitations

## Discussion

The main findings of this study were that the types of symptoms experienced by LC patients affect their adoption of exercise, dietary behavior, and QOL. Experiencing SOB was associated with less readiness for change in adopting exercise, experiencing nausea and vomiting was associated with more readiness for change in adopting exercise, and experiencing RUQ pain was associated with more readiness for change in consuming F&V. In addition, experiencing muscle cramps, anorexia, RUQ pain and body pain, itching, ascites or edema, bruising, and change in appearance negatively affected QOL of LC patients.

Experiencing SOB was associated with less readiness for change in engaging in exercise. In patients with cirrhosis, SOB can be complicated by ascites or pleural effusion. Dyspnea is well known to induce exercise intolerance, and individuals with daily-life dyspnea have more limited exercise capacity [29]. SOB may have inhibited exercise in the cirrhosis patient population because this symptom becomes worse during exercise. Patients who perceive a temporary worsening of symptoms during physical activity may be less physically active [30]. Previous studies of the relationship between dyspnea and exercise intolerance were conducted in patients with pulmonary disease [29] and multiple sclerosis [30]. Therefore, further study is required to determine the impact of SOB on exercise levels in LC patients.

We found that experiencing RUQ pain correlated with more readiness for change in F&V consumption. According to the Health Belief Model, patients will perform healthy behaviors if they believe there is a high risk that the symptoms and complications could adversely affect their lives. The presence of symptoms may prompt patients to adopt healthy behaviors. On the other hand, it can be interpreted that abdominal pain can occur due to certain food intolerance [20]. Patients who believe that their abdominal pain is related to food intolerance may restrict the diet, possibly causing nutritional deficiencies and sarcopenia [31]. Thus, it should be clinically recognized whether the presence of abdominal pain is associated with a healthy diet or different clinical scenarios (i.e., food allergy and intolerance, functional gastrointestinal disorder). In addition, a prospective study design is required to examine the causal relationship between the presence of symptoms and healthy diet behaviors.

The association between the development of nausea and more readiness for change in engaging in exercise may be because exercise can induce gastrointestinal symptoms, such as nausea, heartburn, and abdominal pain [32]. This is because exercise can increase the level of catecholamine, which activates adrenergic receptors and induces nausea and vomiting [33]. In contrast, most longitudinal studies of patients with cancer have reported that those who exercise experienced significantly less intense nausea and greater alleviation of other symptoms [34]. Because the current study had a cross-sectional design, we cannot determine whether nausea led to increased exercise or if increased exercise led to nausea. However, it is unlikely that patients with LC would exercise so intensely as to cause gastrointestinal symptoms. Thus, we believe that the experience of gastrointestinal symptoms was a cue that increased SOC in engaging in exercise, according to the Health Belief Model. Further longitudinal studies are required to find out the causal relationship between symptoms and the adoption of exercise behaviors.

Experiencing muscle cramps can have a negative influence on physical functioning and increase role limitations. Patients with LC typically describe muscle cramps as abrupt, uncomfortable squeezing or contraction of a muscle that lasts seconds to minutes [35]. The finding supports previous results, which reported that muscle cramps are closely associated with the perception of poor health status among patients with LC [17], and that muscle cramps are related to significantly decreased QOL in patients with LC [36]. Muscle cramps are intermittent and difficult to measure using a diagnostic tool, thus clinicians often overlook this symptom [36]. However, they can significantly affect the QOL of patients with LC; thus, professional clinical nurses should help in the assessment of this symptom.

Anorexia can negatively affect physical and social functioning and increase role limitations. This finding was consistent with previous studies, which reported that patients with LC and gastrointestinal symptoms had profound reductions in physical functioning, based on SF-36 scores [37], and that malnutrition due to anorexia was significantly associated with self-perception of lower QOL in patients with LC [38].

RUQ pain and body pain can also negatively affect physical and social functioning. Although pain is not among the most serious symptoms, a small change in patients’ pain experience may significantly affect QOL; thus, it is important that professional clinical nurses carefully assess each patient’s individual experience of pain.

Itching and ascites/edema had a negative impact on physical functioning. Pruritus is an extrahepatic symptom that is the greatest burden for patients with LC. In patients with LC, persistent itching can lead to severe sleep loss, depression, and suicidal thoughts [39]. Based on the reports of previous studies, ascites, serum sodium levels, and lower extremity edema were independently associated with impaired physical functioning [40] in patients with LC. Thus, professional clinical nurses should perform systematic assessments of patients with LC who present with itching, ascites, and edema.

Role limitations due to emotional problems were greater in those who experienced changes in appearance. We speculate that a patient with LC who has readily visible symptoms (itching, edema, and ascites) may experience greater problems with body self-image and consequently greater emotional distress. Previous clinical studies of patients with cancer reported that changes in physical appearance that resulted from disease progression or treatment produce psychological distress [41, 42]. The development of changes in appearance can have a profound impact on multiple functions such as working with others [43]. The present study of patients with LC indicated that emotional problems due to a poor body self-image seemed to be associated with greater role limitations. When referring a patient with LC for psychosocial care, health care professionals should be aware of a patient’s body self-image as a potential indicator of poor role functioning.

This study indicated that symptoms had an impact on QOL, yet higher SOC in engaging exercise and consumption of F&V did not. Although some studies have reported an exercise-induced improvement in the metabolic profile [44], a randomized controlled trial on home-based physical activity and diet intervention showed no QOL improvement [45]. Thus, more evidence based on prospective or experimental studies that supports the benefits of exercise and diet in relation to cirrhosis is still needed, with emphasis on individuals with cardiovascular risk, musculoskeletal disorders, and complications related to cirrhosis. Cirrhosis patients require certified exercise and diet professionals who can perform a detailed functional assessment and design an individualized exercise and diet regimen to improve their QOL [6].

The major limitations of this study are the use of a cross-sectional design and the small sample size. However, because many previous studies on this topic also examined about 100 individuals, we believe it is reasonable to compare our results with those of previous studies. In addition, our results suggest that the adoption of healthy behaviors had no significant effect on QOL. Therefore, further longitudinal or experimental studies on patients with LC are needed to identify the causal relationship between the adoption of healthy behaviors and QOL. This study should be a starting point for a prospective study design (cohort or experimental ones) to confirm whether healthy living habits have a long-term impact on the QOL of patients with cirrhosis. Despite these limitations, very few previous studies have identified the various types of symptoms and QOL in patients with LC.

## Conclusions

In conclusion, this study of LC patients indicated that the type of symptoms experienced by a patient can lead to the adoption or rejection of healthy behaviors and that the patient symptoms can also negatively affect QOL. Patients with LC require specialized support for the assessment and management of symptoms. Identification of the association between symptoms with the intention of adopting healthy behaviors and QOL provides a basis for the development of symptom management strategies and other interventions that may improve the QOL of patients with LC.

## Data Availability

The data sets generated during and/or analyzed during the current study are available from the corresponding authors on reasonable request.

## Abbreviations

LC: Liver cirrhosis
F&V: Fruits and vegetables
QOL: Quality of life
TTM: Transtheoretical model
SOC: Stage of change
RUQ: Right upper quadrant
SOB: Shortness of breath
SF-36: Short-Form Health Survey-36
aOR: Adjusted odds ratio
CI: Confidence intervals
SD: Standard deviation
M: Mean

## References

[1] World Health Organization: Global Hepatitis Report; 2017.

[2] Wittmer VL, Lima RT, Maia MC, Duarte H, Paro FM (2020). Respiratory and symptomatic impact of ascitis relief by paracentesis in patients with hepatic cirrhosis. Arq Gastroenterol.

[3] Alavinejad P, Hajiani E, Danyaee B, Morvaridi M (2019). The effect of nutritional education and continuous monitoring on clinical symptoms, knowledge, and quality of life in patients with cirrhosis. Gastroenterol Hepatol Bed Bench.

[4] Macdonald S, Jepsen P, Alrubaiy L, Watson H, Vilstrup H, Jalan R (2019). Quality of life measures predict mortality in patients with cirrhosis and severe ascites. Aliment Pharmacol Ther.

[5] Les I, Doval E, Flavia M, Jacas C, Cardenas G, Esteban R, Guardia J, Cordoba J (2010). Quality of life in cirrhosis is related to potentially treatable factors. Eur J Gastroenterol Hepatol.

[6] Tandon P, Ismond KP, Riess K, Duarte-Rojo A, Al-Judaibi B, Dunn MA, Holman J, Howes N, Haykowsky MJF, Josbeno DA, McNeely M. Exercise in cirrhosis: Translating evidence and experience to practice. *J Hepatol.* 2018. 10.1016/j.jhep.2018.06.017 29964066

[7] Barnes RY, Jelsma J, Parker R (2019). Improvements in health-related quality of life and function in middle-aged women with chronic diseases of lifestyle after participating in a non-pharmacological intervention programme: a pragmatic randomised controlled trial. Afr J Disabil.

[8] Chen HW, Dunn MA (2018). Arresting frailty and sarcopenia in cirrhosis: future prospects. Clin Liver Disease.

[9] Macias-Rodriguez RU, Ruiz-Margain A, Roman-Calleja BM, Moreno-Tavarez E, Weber-Sangri L, Gonzalez-Arellano MF, Fernandez-Del-Rivero G, Ramirez-Soto K (2019). Exercise prescription in patients with cirrhosis: recommendations for clinical practice. Rev Gastroenterol Mex.

[10] Buscail C, Bourcier V, Fezeu LK, Roulot D, Brule S, Ben-Abdesselam Z, Cagnot C, Hercberg S, Nahon P, Ganne-Carrie N, Julia C. Eating patterns in patients with compensated cirrhosis: a case-control study. *Nutrients.* 2018;10. 10.3390/nu10010060 PMC5793288 29320416

[11] Jones JC, Coombes JS, Macdonald GA (2012). Exercise capacity and muscle strength in patients with cirrhosis. Liver Transpl.

[12] Gundling F, Seidl H, Pehl C, Schmidt T, Schepp W (2009). How close do gastroenterologists follow specific guidelines for nutrition recommendations in liver cirrhosis? A survey of current practice. Eur J Gastroenterol Hepatol.

[13] Campollo O, Sprengers D, Dam G, Vilstrup H, McIntyre N (2017). Protein tolerance to standard and high protein meals in patients with liver cirrhosis. World J Hepatol.

[14] Prochaska JO, Velicer WF (1997). The transtheoretical model of health behavior change. Am J Health Promot.

[15] Prochaska JO, Velicer WF, Rossi JS, Goldstein MG, Marcus BH, Rakowski W, Fiore C, Harlow LL, Redding CA, Rosenbloom D (1994). Stages of change and decisional balance for 12 problem behaviors. Health Psychol.

[16] Adinolfi LE, Gambardella M, Andreana A, Tripodi M-F, Utili R, Ruggiero G (2001). Steatosis accelerates the progression of liver damage of chronic hepatitis C patients and correlates with specific HCV genotype and visceral obesity. Hepatology.

[17] Marchesini G, Bianchi G, Amodio P, Salerno F, Merli M, Panella C, Loguercio C, Apolone G, Niero M, Abbiati R. Factors associated with poor health-related quality of life of patients with cirrhosis. *Gastroenterology.* 2001;120:170-8 10.1053/gast.2001.21193 11208726

[18] Williams FR, Berzigotti A, Lord JM, Lai JC, Armstrong MJ (2019). Review article: impact of exercise on physical frailty in patients with chronic liver disease. Aliment Pharmacol Ther.

[19] Casales da Silva Vieira R, Álvares-da-Silva MR, de Oliveira ÁR, da Silveira GJ, Kruger RL, Dal Bosco A, Marroni NAP, Forgiarini LAJ, Dias AS (2018). Cirrhosis affects maximal oxygen consumption, functional capacity, quality of life in patients with hepatitis C. Physiother Res Int.

[20] Aamann L, Dam G, Borre M, Drljevic-Nielsen A, Overgaard K, Andersen H, Vilstrup H, Aagaard NK (2020). Resistance training increases muscle strength and muscle size in patients with liver cirrhosis. Clin Gastroenterol Hepatol.

[21] Zhang X, Xi W, Liu L, Wang L (2019). Improvement in quality of life and activities of daily living in patients with liver cirrhosis with the use of health education and patient health empowerment. Med Sci Monit.

[22] Muto Y, Sato S, Watanabe A, Moriwaki H, Suzuki K, Kato A, Kato M, Nakamura T, Higuchi K, Nishiguchi S, Kumada H (2005). Effects of oral branched-chain amino acid granules on event-free survival in patients with liver cirrhosis. Clin Gastroenterol Hepatol.

[23] Lemyze M, Dharancy S, Nevière R, Wallaert B (2011). Cardiopulmonary response to exercise in patients with liver cirrhosis and impaired pulmonary gas exchange. Respir Med.

[24] Peng JK, Hepgul N, Higginson IJ, Gao W (2019). Symptom prevalence and quality of life of patients with end-stage liver disease: a systematic review and meta-analysis. Palliat Med.

[25] Kim SH, Oh EG, Lee WH (2006). Symptom experience, psychological distress, and quality of life in Korean patients with liver cirrhosis: a cross-sectional survey. Int J Nurs Stud.

[26] Briggs Early K, Armstrong Shultz J, Evans M, Corbett CF, Nicholson Butkus S, Massey L (2012). Dietary goal attainment measures and psychosocial factors among Mexican Americans and non-Hispanic whites with type 2 diabetes. Ecol Food Nutr.

[27] Brown JK, Byers T, Doyle C, Coumeya KS, Demark-Wahnefried W, Kushi LH, McTieman A, Rock CL, Aziz N, Bloch AS (2003). Nutrition and physical activity during and after cancer treatment: an American Cancer Society guide for informed choices. CA Cancer J Clin.

[28] Kim SH, Jo MW, Lee SI (2013). Psychometric properties of the Korean short form-36 health survey version 2 for assessing the general population. Asian Nurs Res (Korean Soc Nurs Sci).

[29] Rocha A, Arbex FF, Sperandio PA, Souza A, Biazzim L, Mancuso F, Berton DC, Hochhegger B, Alencar MCN, Nery LE (2017). Excess ventilation in chronic obstructive pulmonary disease-heart failure overlap. implications for dyspnea and exercise intolerance. Am J Respir Crit Care Med.

[30] Moumdjian L, Gervasoni E, Van Halewyck F, B OE, Wens I, Van Geel F, Van Wijmeersch B, Feys P, Van Asch P: Walking endurance and perceived symptom severity after a single maximal exercise test in persons with mild disability because of multiple sclerosis. *Int J Rehabil Res.* 2018. 10.1097/MRR.0000000000000305 30020095

[31] Pasqui F, Poli C, Colecchia A, Marasco G, Festi D (2015). Adverse food reaction and functional gastrointestinal disorders: role of the dietetic approach. J Gastrointestin Liver Dis.

[32] Kondo T, Nakae Y, Mitsui T, Kagaya M, Matsutani Y, Horibe H, Read NW (2001). Exercise-induced nausea is exaggerated by eating. Appetite.

[33] King KS, Darmani NA, Hughes MS, Adams KT, Pacak K (2010). Exercise-induced nausea and vomiting: another sign and symptom of pheochromocytoma and paraganglioma. Endocrine.

[34] Lee J, Dodd MJ, Dibble SL, Abrams DI (2008). Nausea at the end of adjuvant cancer treatment in relation to exercise during treatment in patients with breast cancer. Oncol Nurs Forum.

[35] Konikoff F, Theodor E (1986). Painful muscle cramps. A symptom of liver cirrhosis?. J Clin Gastroenterol.

[36] Chatrath H, Liangpunsakul S, Ghabril M, Otte J, Chalasani N, Vuppalanchi R (2012). Prevalence and morbidity associated with muscle cramps in patients with cirrhosis. Am J Med.

[37] Kalaitzakis E, Simren M, Olsson R, Henfridsson P, Hugosson I, Bengtsson M, Bjornsson E (2006). Gastrointestinal symptoms in patients with liver cirrhosis: associations with nutritional status and health-related quality of life. Scand J Gastroenterol.

[38] Rojas-Loureiro G, Servin-Caamano A, Perez-Reyes E, Servin-Abad L, Higuera-de la Tijera F (2017). Malnutrition negatively impacts the quality of life of patients with cirrhosis: an observational study. World J Hepatol.

[39] Hegade VS, Bolier R, Oude Elferink RP, Beuers U, Kendrick S, Jones DE (2016). A systematic approach to the management of cholestatic pruritus in primary biliary cirrhosis. Front Gastroenterol.

[40] Sola E, Watson H, Graupera I, Turon F, Barreto R, Rodriguez E, Pavesi M, Arroyo V, Guevara M, Gines P (2012). Factors related to quality of life in patients with cirrhosis and ascites: relevance of serum sodium concentration and leg edema. J Hepatol.

[41] Galiano-Castillo N, Ariza-Garcia A, Cantarero-Villanueva I, Fernandez-Lao C, Diaz-Rodriguez L, Arroyo-Morales M (2014). Depressed mood in breast cancer survivors: associations with physical activity, cancer-related fatigue, quality of life, and fitness level. Eur J Oncol Nurs.

[42] Reese JB, Handorf E, Haythornthwaite JA (2018). Sexual quality of life, body image distress, and psychosocial outcomes in colorectal cancer: a longitudinal study. Support Care Cancer.

[43] Lee MK, Kang HS, Lee KS, Lee ES (2017). Three-year prospective cohort study of factors associated with return to work after breast cancer diagnosis. J Occup Rehabil.

[44] Naseer M, Turse EP, Syed A, Dailey FE, Zatreh M, Tahan V (2019). Interventions to improve sarcopenia in cirrhosis: a systematic review. World J Clin Cases.

[45] Chen HW, Ferrando A, White MG, Dennis RA, Xie J, Pauly M, Park S, Bartter T, Dunn MA, Ruiz-Margain A, et al. Home-based physical activity and diet intervention to improve physical function in advanced liver disease: a randomized pilot trial. *Dig Dis Sci.* 2020. 10.1007/s10620-019-06034-2 31907774

